# Evaluating residual tumor after neoadjuvant chemotherapy for muscle-invasive urothelial bladder cancer: diagnostic performance and outcomes using biparametric vs. multiparametric MRI

**DOI:** 10.1186/s40644-023-00632-0

**Published:** 2023-11-14

**Authors:** Sungmin Woo, Anton S. Becker, Jeeban P. Das, Soleen Ghafoor, Yuki Arita, Nicole Benfante, Natalie Gangai, Min Yuen Teo, Alvin C. Goh, Hebert A. Vargas

**Affiliations:** 1 Department of Radiology, Memorial Sloan Kettering Cancer Center, 1275 York Avenue, New York, NY 10065 USA; 2 Department of Radiology, NYU Langone Health, 660 1st Avenue, New York, NY 10016 USA; 3 Institute of Diagnostic and Interventional Radiology, University Hospital Zurich, Rämistrasse 100, Zürich, CH-8091 Switzerland; 4 Urology Service, Department of Surgery, Memorial Sloan Kettering Cancer Center, 1275 York Avenue, New York, NY 10065 USA; 5 Genitourinary Oncology Service, Department of Medicine, Memorial Sloan Kettering Cancer Center, New York, NY 10065 USA

**Keywords:** Biparametric, Cystectomy, Magnetic resonance imaging, Muscle-invasive bladder cancer, Multiparametric, Neoadjuvant chemotherapy, Prognosis, Survival, Urothelial, Response assessment

## Abstract

**Background:**

Neoadjuvant chemotherapy (NAC) before radical cystectomy is standard of care in patients with muscle-invasive bladder cancer (MIBC). Response assessment after NAC is important but suboptimal using CT. We assessed MRI without vs. with intravenous contrast (biparametric [BP] vs. multiparametric [MP]) for identifying residual disease on cystectomy and explored its prognostic role.

**Methods:**

Consecutive MIBC patients that underwent NAC, MRI, and cystectomy between January 2000–November 2022 were identified. Two radiologists reviewed BP-MRI (T2 + DWI) and MP-MRI (T2 + DWI + DCE) for residual tumor. Diagnostic performances were compared using receiver operating characteristic curve analysis. Kaplan-Meier curves and Cox proportional-hazards models were used to evaluate association with disease-free survival (DFS).

**Results:**

61 patients (36 men and 25 women; median age 65 years, interquartile range 59–72) were included. After NAC, no residual disease was detected on pathology in 19 (31.1%) patients. BP-MRI was more accurate than MP-MRI for detecting residual disease after NAC: area under the curve = 0.75 (95% confidence interval (CI), 0.62–0.85) vs. 0.58 (95% CI, 0.45–0.70; p = 0.043). Sensitivity were identical (65.1%; 95% CI, 49.1–79.0) but specificity was higher in BP-MRI compared with MP-MRI for determining residual disease: 77.8% (95% CI, 52.4–93.6) vs. 38.9% (95% CI, 17.3–64.3), respectively. Positive BP-MRI and residual disease on pathology were both associated with worse DFS: hazard ratio (HR) = 4.01 (95% CI, 1.70–9.46; p = 0.002) and HR = 5.13 (95% CI, 2.66–17.13; p = 0.008), respectively. Concordance between MRI and pathology results was significantly associated with DFS. Concordant positive (MRI+/pathology+) patients showed worse DFS than concordant negative (MRI-/pathology-) patients (HR = 8.75, 95% CI, 2.02–37.82; p = 0.004) and compared to the discordant group (MRI+/pathology- or MRI-/pathology+) with HR = 3.48 (95% CI, 1.39–8.71; p = 0.014).

**Conclusion:**

BP-MRI was more accurate than MP-MRI for identifying residual disease after NAC. A negative BP-MRI was associated with better outcomes, providing complementary information to pathological assessment of cystectomy specimens.

**Supplementary Information:**

The online version contains supplementary material available at 10.1186/s40644-023-00632-0.

## Introduction

Muscle-invasive bladder cancer (MIBC), albeit localized, is associated with elevated risk for metastatic dissemination and therefore disease-related morbidity and mortality [1]. Radical cystectomy is the gold standard for management of MIBC and the addition of neoadjuvant chemotherapy (NAC) reduces recurrence and improves overall survival, potentially due to eliminate micro-metastases, as demonstrated in multiple prospective clinical trials [2–4]. Pathologic responses response to NAC (e.g., either pathologic complete response or downgrading to < pT2) is associated with reduced recurrence and increased survival [5–7]. However, assessment of NAC response is frequently only achievable after radical cystectomy from extensive pathologic evaluation. The ability to predict response prior to radical cystectomy can theoretically identify responders who may benefit from a bladder-sparing approach, and recognize non-responders who may benefit from alternative systemic therapy or upfront surgery.

Assessing treatment response of MIBC using conventional imaging (e.g., computed tomography [CT]) has been regarded as difficult [8]. It is well known that changes related to the treatment itself (e.g., NAC and transurethral resection [TUR]) including inflammation and fibrosis causes false-positive results or conversely obscure residual tumor, resulting in suboptimal diagnostic performance of CT for determining treatment response [9]. Magnetic resonance imaging (MRI) has been gaining momentum for the pretreatment assessment of bladder cancer, especially with regards to determining muscle-invasiveness using a novel standardized approach for performing and interpreting MRI called Vesical Imaging and Reporting Data System (VI-RADS) [10, 11]. This stems from the powerful capability of MRI to provide a combination of superior soft tissue resolution on anatomical imaging (e.g., T2-weighted imaging [T2WI]) along with functional imaging (e.g., diffusion-weighted imaging [DWI] and dynamic contrast-enhanced [DCE] MRI). Nevertheless, it has not been well established whether this advantage of MRI will allow it to be the preferred modality for determining residual muscle invasiveness in a NAC-treated bladder, in addition to treatment response and if so, what the optimal imaging method for post-treatment interpretation of MRI will be. For example, it is unclear if the full comprehensive set of MRI sequences using intravenous (IV) contrast administration (i.e., “*multiparametric*” [MP]) is superior to a shortened protocol using only T2WI and DWI and without IV contrast (i.e., “*biparametric*” [BP]), which has been universal asked in various types of pelvic malignancies [12–14].

The purpose of this study was to compare the diagnostic performance of MP- and BP-MRI in the determination of residual disease in NAC-treated MIBC prior to radical cystectomy. In addition, we aimed to assess the prognostic value of imaging response, and whether it provides complementary information to pathological response.

## Materials and methods

### Study population

**Fig. 1:**
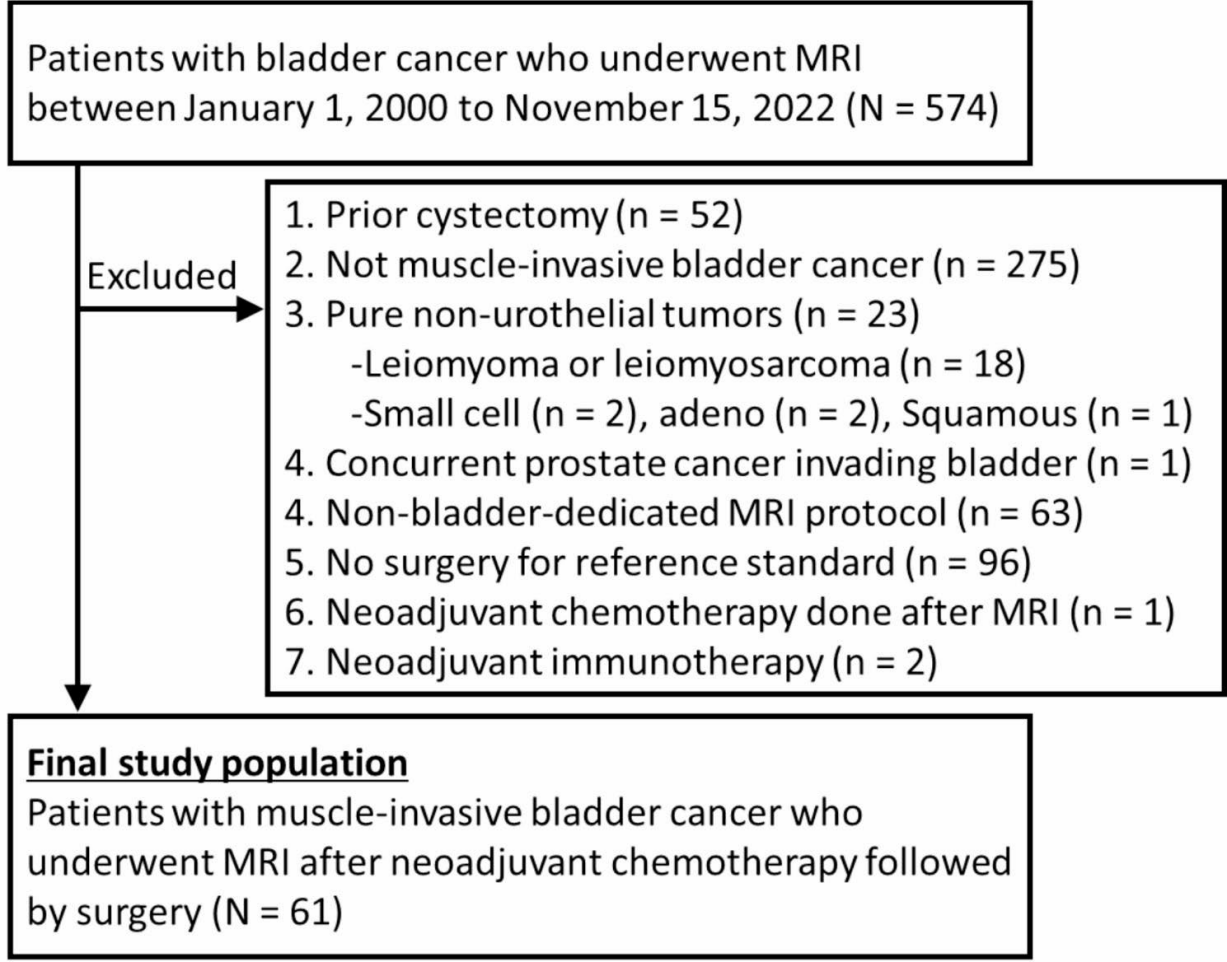
Flowchart for patient selection

This retrospective study was performed after obtaining approval from the institutional review board and was compliant with the Health Insurance Portability and Accountability Act. Due to the retrospective nature, the need for informed consent was waived. Our electronic medical records (EMR) and radiology information system databases were searched to identify patient who underwent MRI for MIBC (cT2–4NxM0) between January 1, 2000 and November 15, 2022. Exclusion criteria were: prior cystectomy, pure non-urothelial histology (e.g., spindle cell neoplasms, small cell carcinoma, squamous cell carcinoma, or adenocarcinoma), no chemotherapy prior to MRI, non-dedicated MRI for bladder assessment (e.g., musculoskeletal protocol), and lack of reference standard (i.e., no pathological analysis from cystectomy specimens). Figure 1 summarizes the flow chart for patient selection.

### Imaging assessments

All MRI examinations were performed on one of 1.5- or 3.0-Tesla MRI scanners (GE Healthcare, Chicago IL) at our institution. Owing to the long study period, there was variability in the detailed technical parameters; however, the multiparametric MRI protocol generally consisted of tri-planar small field-of-view T2WI focused on the bladder, large field-of-view axial T1-weighted imaging (T1WI), DWI with b-values up to 700 s/mm^2^, and DCE MRI after intravenous injection of gadolinium-based agents as described in prior publications [15, 16]. Imaging interpretation was performed by two radiologists specialized in genitourinary oncologic imaging (H.A.V. and S.W., with 12 and 3 years of post-fellowship experience, respectively) in consensus, who were aware that the patients had bladder cancer but were blinded to other clinical and pathology information. In order to compare the diagnostic performance of BP- and MP-MRI for determining residual tumor, a stepwise approach was taken [17]: first, only the T2WI and DWI were jointly assessed (i.e., BP-MRI) followed by additional provision of DCE MRI (i.e., MP-MRI). Each patient’s MRI was graded on a 5-point Likert scale for each of the two steps, regarding the likelihood of residual tumor based on findings reported in the literature. For example, focal nodular or diffuse irregular wall thickening with intermediate signal intensity on T2WI, high signal intensity foci corresponding to this region on high b-value DWI, and early enhancement on DCE MRI were considered to represent residual tumor [5, 17–20]. A score of 1 was given if none of these findings were present and 5, if all of these findings were unequivocally present. Scores of 2 through 4 were assigned when these findings were considered to be intermediate between these two extreme scenarios.

### Clinicopathological information

The EMR was searched to identify the following information: age, gender, clinical stage, details of neoadjuvant chemotherapy (e.g., type of drug and number of cycles), details of surgery (e.g., radical vs. partial cystectomy), histological information, pathologic stage, and follow-up information (last date, recurrence, metastasis, and survival).

### Statistical analysis

Quantitative data are presented as medians with range or means with their standard deviations; categorical data as frequency and percentages. Receiver operating characteristic (ROC) curve analysis was used to assess the diagnostic performance of MRI for determining residual cancer based on pathological analysis of the cystectomy specimens as the reference standard. Microscopic foci of tumor on cystectomy specimens were considered positive for residual disease on pathology. The area under the ROC curves (AUC) were compared between BP- and MP-MRI assessments. For the purpose of analysis, MRI Likert scale scores of 4 and 5 were considered positive for residual tumor and 1, 2, and 3 as negative and the corresponding sensitivity, specificity, and positive predictive value were calculated for determining residual disease on pathology. Additionally, diagnostic performance of BP- and MP-MRI were assessed for determining residual MIBC (vs. non-MIBC) on pathology using the same MRI interpretations for determining residual disease. For the remainder of the analyses, we planned to use the best approach (i.e., biparametric vs. multiparametric) for MRI assessments. For outcomes analyses, patients were classified into one of three groups according to concordance or discordance between MRI and pathology regarding the presence of residual cancer based on prior literature [21]: (1) no residual cancer on both (i.e., concordant negative group), (2) residual cancer on imaging but not on pathology or vice versa (i.e., discordant group), and residual cancer on both (i.e., concordant positive group). Disease-free survival (DFS) was defined as the time from cystectomy to that of recurrence, metastasis or death [5, 22] and was estimated using the Kaplan-Meier method. Differences in DFS between the groups were compared with the Cox proportional-hazards models and the log-rank test with Bonferroni correction to account for multiple comparisons. Statistical analyses were performed using statistical software R (version 4.3.0, Vienna, Austria). A p-value of < 0.05 was considered to be statistically significant.

## Results

### Patient and clinicopathological characteristics

The patient and clinicopathological characteristics are summarized in Table 1. Sixty-one patients, 36 men (59.0%) and 25 women (41.0%) with median age of 65 years (interquartile range [IQR] 59 − 72) were included. Clinical stages were cT2 in 27 (44.3%), cT3 in 20 (32.8%) and cT4 in 14 (23.0%). All but 6 (9.8%) received 4 or more cycles of NAC which were mostly cisplatin-based (n = 54 [88.5%]) and the remaining carboplatin-based (n = 7 [11.5%]). The median time from completion of NAC to MRI was 14 days (IQR 7–23). Patients underwent partial (n = 4 [6.6%]) or radical cystectomies (n = 57 [93.4%]) after a median of 33 days from the MRI (IQR 24–42). At cystectomy, there was no residual disease (ypT0) in 19 patients (31.1%). Forty-two patients (68.9%) had residual disease, among which 27 were muscle-invasive (ypT2, n = 7 [11.5%]; pT3, n = 15 [24.6%]; and ypT4, n = 5 [8.2%]). 15 of these 42 were microscopic residual disease. Thirty-four patients (55.7%) had pure urothelial cancer and 27 (44.3%) had urothelial cancer with variants. After a median of 461 days (IQR 162 − 1048) of follow-up, 23 patients experienced recurrence after surgery (among which 17 died), 5 patients died without disease, and the remaining 33 patients were alive without evidence of disease.

**Table 1:** Clinical and pathological characteristics

Clinicopathological variable		Number (%) *
Age (years)		65 (59–72)
Gender	Male	36 (59.0%)
	Female	25 (41.0%)
Clinical T stage	cT2	27 (44.3%)
	cT3	20 (32.8%)
	cT4	14 (23.0%)
Histological subtype	NOS	34 (55.7%)
	NOS with glandular	2 (3.3%)
	NOS with lymphoepithelioma-like	1 (1.6%)
	NOS with microcystic	1 (1.6%)
	NOS with micropapillary	2 (3.3%)
	NOS with nested	1 (1.6%)
	NOS with plasmacytoid	3 (4.9%)
	NOS with sarcomatoid	3 (4.9%)
	NOS with small cell	6 (9.8%)
	NOS with squamous	8 (13.1%)
Type of neoadjuvant chemotherapy	Gemcitabine + cisplatin	40 (65.6%)
	Gemcitabine + cisplatin + paclitaxel	6 (9.8%)
	Gemcitabine + carboplatin	3 (4.9%)
	Gemcitabine + carboplatin + paclitaxel	2 (3.3%)
	Methotrexate + vinblastine + doxorubicin + cisplatin ^†^	4 (6.6%)
	Etoposide + Cisplatin ^‡^	4 (6.6%)
	Etoposide + Carboplatin	2 (3.3%)
Type of surgery	Partial cystectomy	4 (6.6%)
	Radical cystectomy	57 (93.4%)
Pathological staging on cystectomy	ypT0	19 (31.1%)
	ypTa	2 (3.3%)
	ypTis	6 (9.8%)
	ypT1	7 (11.5%)
	ypT2	7 (11.5%)
	ypT3	15 (24.6%)
	ypT4	5 (8.2%)
Time interval (days)	Neoadjuvant chemotherapy to MRI	14 (7–23)
	MRI to cystectomy	33 (24–42)
	Cystectomy to follow-up	461 (162–1048)

MRI = magnetic resonance imaging; NOS = not otherwise specified

* Number of patients with percentages in parentheses for categorical variables and medians with interquartile ranges (IQR) in parentheses for continuous variables

^†^ 3 of 4 with dose-dense regimen

^‡^ 1of 4 with Durvalumab

### Diagnostic performance of MRI for determining residual disease

**Fig. 2:**
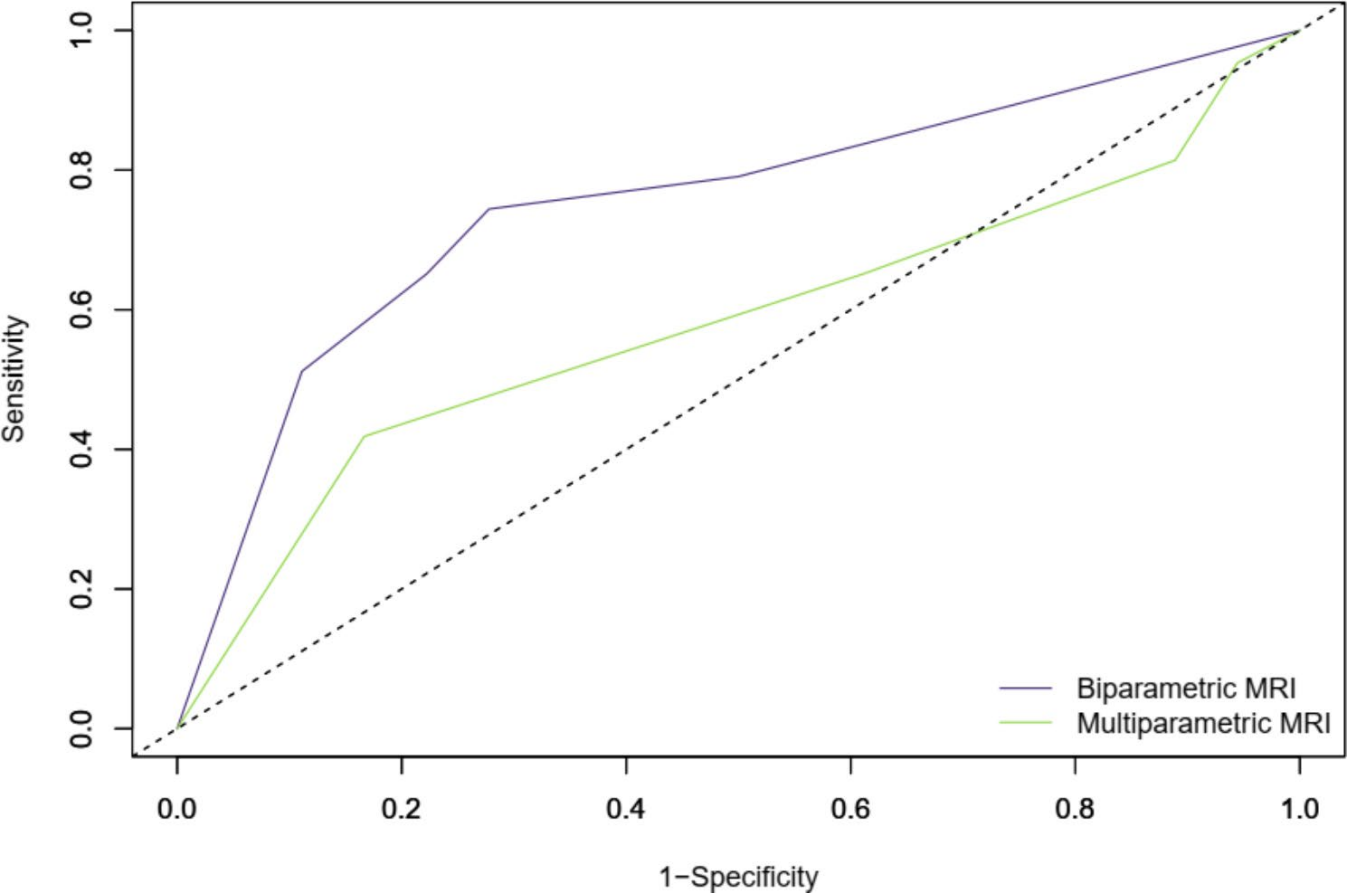
Receiver operating characteristic (ROC) analysis comparing diagnostic performance of biparametric and multiparametric MRI for detecting residual tumor on cystectomy specimens after neoadjuvant chemotherapy. Biparametric MRI was superior to multiparametric MRI with corresponding areas under the ROC of 0.75 (95% confidence interval [CI], 0.62–0.88) and 0.58 (95% CI, 0.44–0.72), respectively

**Fig. 3:**
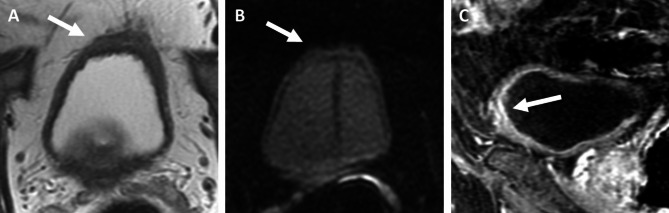
Seventy-two-year old man with high grade muscle-invasive urothelial bladder cancer on transurethral resection. MRI performed after 3 cycles of neoadjuvant gemcitabine + cisplatin, showed residual focal wall thickening (arrow) in the anterior bladder wall on axial T2-weighted imaging (**A**). There was no abnormal high signal on axial diffusion-weighed imaging (**B**) but mild ill-defined enhancement was noted on sagittal post-contrast MRI (**C**). At radical cystectomy, there was a 0.5-cm focus of residual tumor in situ (ypTis). Patient was alive without recurrence at 792 days after surgery

**Fig. 4:**
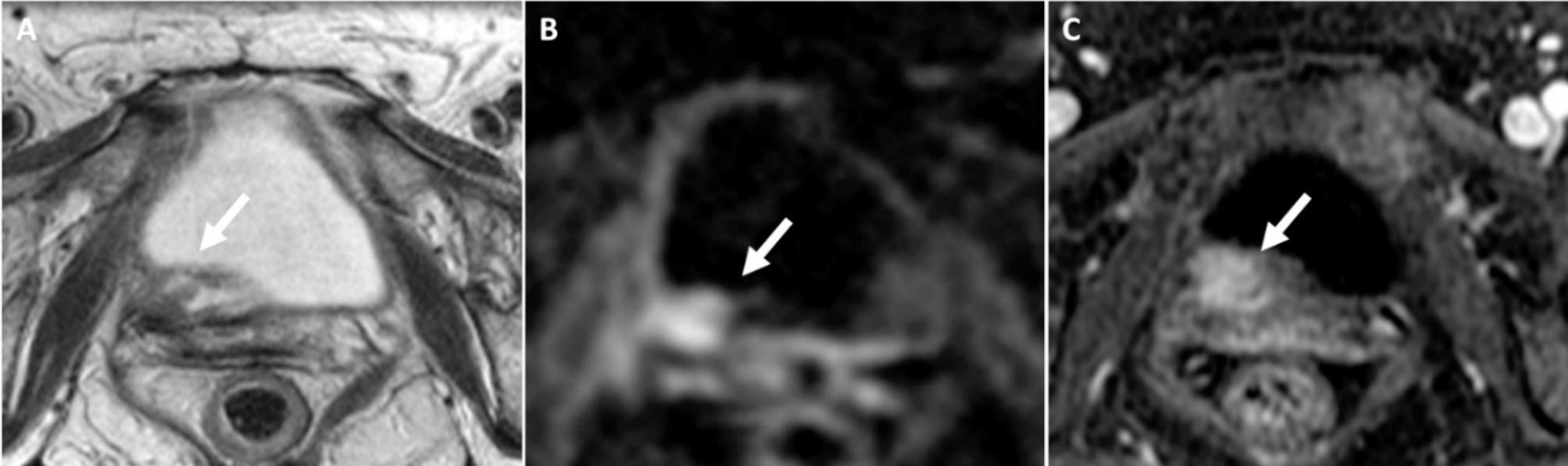
Fifty-five-year old woman with high grade muscle-invasive urothelial bladder cancer on transurethral resection. MRI performed after 4 cycles of neoadjuvant gemcitabine + carboplatin, showed an intermediate signal nodule (arrow) on T2-weighted imaging (**A**) with corresponding focal high signal on diffusion-weighed imaging (**B**) and early and strong enhancement on post-contrast MRI (**C**). At radical cystectomy, there was residual tumor with perivesical fat invasion (ypT3). Tumor recurred at the pelvic sidewall at 78 days after surgery and the patient subsequently died at 128 days after surgery

**Fig. 5:**
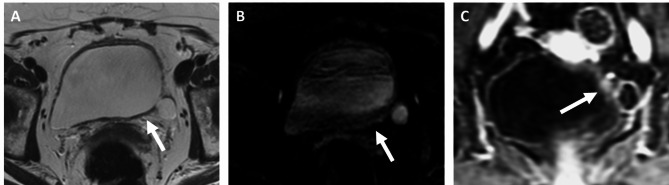
Sixty-four-year old woman with high grade muscle-invasive urothelial bladder cancer and small cell variant histology on transurethral resection. MRI performed after 4 cycles of neoadjuvant etoposide + cisplatin, showed minimal thickening on axial T2-weighted imaging at the site of resected tumor (**A**), no increased signal on axial diffusion-weighted imaging (**B**) but focal early nodular enhancement on sagittal post-contrast MRI (**C**). This was considered a negative biparametric and positive multiparametric MRI. At radical cystectomy, there was no residual tumor (ypT0). Patient was alive without recurrence at 1048 days after surgery

BP-MRI was positive for residual tumor in 32 patients (Likert scales of 4 and 5; n = 8 and 24, respectively) and negative in 29 patients (Likert scales of 1, 2, and 3; n = 18, 6, and 5, respectively). MP-MRI was positive for residual tumor in 39 patients (Likert scales of 4 and 5; n = 18 and 21, respectively) and negative in 22 patients (Likert scales of 1, 2, and 3; n = 3, 7, and 12, respectively). The ROC curves showing diagnostic performance of BP- and MP-MRI for determining residual disease and residual MIBC are shown in Fig. 2 and supplementary Fig. 1. The diagnostic performance of BP-MRI was significantly higher than that of MP-MRI with corresponding AUCs of 0.75 (95% confidence interval [CI], 0.62–0.85) and 0.58 (95% CI, 0.45–0.70), respectively (p = 0.043). The sensitivity, specificity, and positive predictive value of BP-MRI for determining the presence of residual disease were 65.1% (28/43; 95% CI, 49.1–79.0), 77.8% (14/18; 95% CI, 52.4–93.6), and 87.5% (28/32; 95% CI, 71.0–96.5) whereas those for MP-MRI were 65.1% (28/43; 95% CI, 49.1–79.0), 38.9% (7/18; 95% CI, 17.3–64.3), and 71.8% (28/39; 95% CI, 55.1–85.0). Among the 15 patients where MRI failed to detect residual disease, the disease was microscopic on the cystectomy specimen in 10 (66.7%) for BP-MRI and 9 (60.0%) for MP- MRI. Representative examples of patients that had different combinations of MRI and pathological findings are shown in Figs. 3, 4 and 5.

### Residual Disease on MRI and pathology, and their association with disease-free survival

**Fig. 6:**
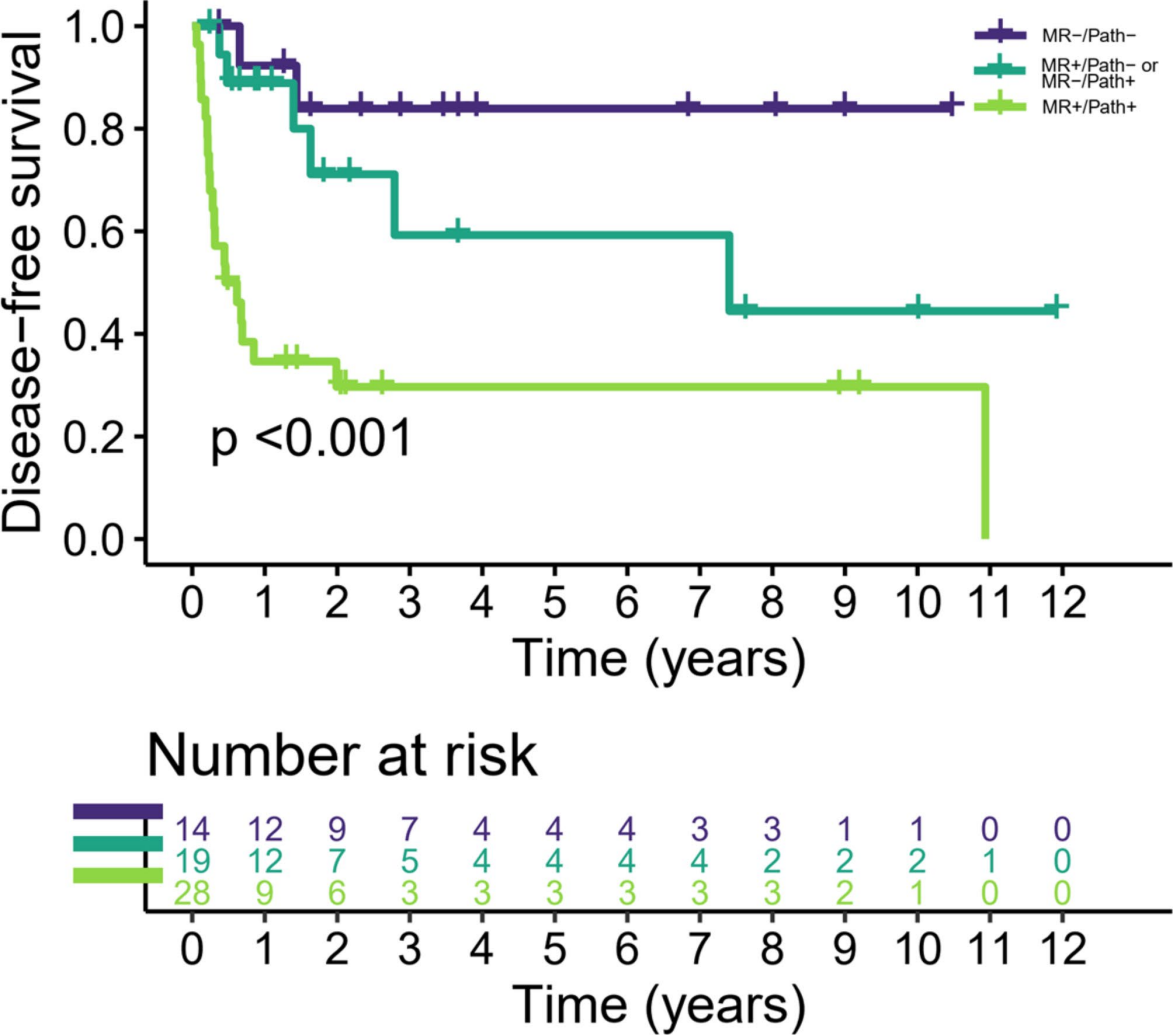
Kaplan-Meier survival curves for disease-free survival in patients who received neoadjuvant chemotherapy followed by cystectomy. The survival estimates between three subgroups based on concordance and discordance between MRI and pathology showed significant difference (p < 0.001)

A positive BP-MRI suspicious for residual disease was significantly associated with worse DFS with a HR of 4.01 (95% CI, 1.70–9.46; p = 0.002). Residual disease on cystectomy specimen was also significantly associated with worse DFS with a HR of 5.13 (95% CI, 2.66–17.13; p = 0.008). Survival curves stratified to MRI and pathology are shown in Fig. 6 and supplementary Fig. 2. When stratifying the patients into 3 groups based on the concordance and discordance between MRI and pathology, there was a significant difference between these groups in their DFS (p < 0.001). Specifically, the concordant positive group (suspicious MRI findings and residual disease on pathology; n = 28) showed significantly worse DFS compared with the concordant negative group (no suspicious MRI findings and no residual disease on pathology; n = 14) with a HR of 8.75 (95% CI 2.02–37.82; p = 0.004) and compared with the discordant group (n = 19 among which 15 had a negative MRI but residual disease on pathology whereas 4 had a suspicious MRI but no residual disease on pathology) with a HR of 3.48 (95% CI 1.39–8.71; p = 0.014). There was no significant difference in DFS between the discordant group and the concordant negative group (HR = 2.51, 95% CI 0.50–12.49; p = 0.524).

## Discussion

In the current study we assessed the diagnostic performance and prognostic role of MRI after neoadjuvant chemotherapy in patients with muscle-invasive urothelial bladder cancer. We found that the MRI after chemotherapy showed a moderately high specificity for determining the presence of residual disease at cystectomy using the *biparametric* approach – that is, performing MRI without IV contrast, performing significantly better than the multiparametric technique. A negative MRI was significantly associated with better DFS after cystectomy, further allowing better risk stratification of patients comprehensively using pathology and MRI. Overall, our findings support the use of MRI in the post-treatment assessment of MIBC for assessing treatment response and prognostication, which will help plan management and counsel patients especially in this era of personalized treatment and increased use of potential bladder-sparing approaches.

The overall diagnostic performance of BP-MRI was moderate (AUC = 0.75) for detecting residual cancer on cystectomy specimens in patients that had underwent neoadjuvant chemotherapy. Known limitations are likely impediments for achieving higher diagnostic accuracies, namely diffuse post-treatment changes, either from the TUR or effects of chemotherapeutic agents which inevitably results in fibrosis and inflammation. These can show abnormal MRI signal changes that may either mimic tumor or obscure the presence of residual tumor and therefore undermining sensitivity and specificity, especially on anatomical MRI sequences such as T2WI. For the same reason, it is recommended that VI-RADS assessment of muscle invasiveness in bladder tumors, should be performed in the pre-treatment setting and preferentially recommended to be performed before a therapeutic TUR. In order to overcome these limitations, using functional sequences has been emphasized for assessing bladder tumors, such as DWI which exploits tissue characteristics such as cellularity or histological aggressiveness in various cancers [23, 24]. Utilizing the advantages of DWI helps improve the specificity of detecting residual tumor after NAC (77.8% in our study) when using T2WI and DWI in conjunction. Nevertheless, detection of microscopic tumor is below the resolution of even DWI and limits sensitivity (66.7% in our study). This is substantiated by the fact that in our study, in 10 of the 15 patients where MRI failed to detect residual disease, the tumors were present only as microscopic foci. In the recent years, there have been attempts to further increase the diagnostic performance of MRI for detecting residual tumor by using quantitative MRI metrics (e.g., apparent diffusion coefficient values on DWI) – however these metrics are highly dependent on technical parameters such as MRI magnet strength, vendor and acquisition parameters and are yet not considered to be universally applicable and therefore warrants further investigation [20, 25].

The conundrum of whether to use IV contrast or not (or in other words, to perform BP-MRI vs. MP-MRI) in the assessment of various malignancies originally stemmed from the rationale that omitting IV contrast administration can save acquisition time, increase patient comfort and access to MRI, and decrease risk of contrast-related adverse reactions while maintaining diagnostic performance [12–14]. In our study, we found that BP-MRI actually was actually more accurate than MP-MRI (AUC = 0.75 vs. 0.58, respectively; p = 0.049). While sensitivity between the two approaches were virtually identical, there was a substantial difference in the specificity: 77.8% vs. 38.9%, respectively. Our results are in agreement with the preliminary observations by Yoshida et al. [19] where the investigators reported a similarly striking difference: specificity of 92% for DWI and 18% for DCE MRI in patients with MIBC that received chemoradiotherapy. Additionally, in the PURE-01 trial, where patients with MIBC underwent neoadjuvant immunotherapy with Pembrolizumab, investigators found that there was no difference in biparametric and multiparametric MRI (AUC 0.74 and 0.74, respectively) with identical sensitivity and specificity for both approaches (0.76 and 0.70, respectively) [26]. The difference of the results from the PURE-01 trial and the above (our study and Yoshida et al. [19]) may stem from differences in the imaging assessment criteria and the systemic therapeutic agent used (chemotherapy vs. immunotherapy). Altogether, these findings are consistent with clinical observations where early enhancement in the area of the tumor is common (which was the diagnostic criteria used in our and other studies) regardless of the presence of residual tumor due to reactive changes, inflammation, and fibrosis and as a result, does not really help determine whether there is residual tumor or not. Based on the collective results of our study and others, adding IV contrast (e.g., MP-MRI) may not only fail to provide incremental value, but even be detrimental for the assessment of residual disease after neoadjuvant treatment. Interestingly, there is also emerging evidence from the pretreatment setting showing that BP-MRI may perform similarly to MP-MRI for determining muscle invasion of bladder tumors [13, 27].

In our study, in addition to assessing the diagnostic performance of MRI for detecting residual tumor after NAC, we evaluated if the potential prognostic role for MRI. First, we found that a positive MRI after NAC was associated with a worse DFS (HR = 3.84, 95% CI 1.64–9.03; p < 0.001) along with residual disease on pathology (HR = 4.99, 95% CI 1.50–16.60; p = 0.004). This corroborates results from a trial where patients with MIBC received NAC with a regimen of dose-dense methotrexate, vinblastine, doxorubicin, and cisplatin by Choueiri et al. [5], where radiological response (using a criteria of > 50% decrease in product of the longest perpendicular diameters) was shown to have a HR of 4.1 (95% CI 1.3–12.5; p = 0.009). Second, we found that when stratifying the patients into 3 groups based on the concordance and discordance between MRI and pathology, the patients that had both a positive MRI and residual disease on pathology had worse DFS compared with not only [1] patients with a negative MRI and no residual disease on pathology (HR = 8.16, 95% CI 1.89–35.10; p = 0.005) but also compared with [2] patients that had discordant results between MRI and pathology (HR = 3.27, 95% CI 1.31–8.12; p = 0.021). The fact that patients that had a positive MRI and residual disease on pathology showed the worst prognosis is easily understood as they represent those harboring a larger cancer burden and aggressive disease that did not respond well to NAC. Regarding the better prognosis of the discordant group (compared with those with negative MRI and pathology), we speculate that these results were mainly driven by patients that had a negative MRI and positive pathology result (15/19 of the discordant subgroup). As discussed above, it can be intuitively understood that larger tumor volume (therefore better detectable on MRI) will confer a worse outcome compared with tumors that are only microscopically present (and therefore not easily detectable on MRI). Although it is not well understood, a few hypotheses could explain the remaining patients showing the opposite pattern of discordance – that is, a positive MRI but no residual tumor on pathology. As post-NAC MRIs were done prior to the cystectomy, we speculate that larger and slower responding tumors may in fact still have harbored residual tumor at the time of MRI but resolved by the time of cystectomy. This phenomenon has been shown in patients with rectal tumors that underwent chemoradiation followed by surgical resection [28]. A similar concept is reflected in the recently proposed MRI interpretation algorithm for assessing response assessment to systemic therapy in bladder cancer called Neoadjuvant chemotherapy VI-RADS (nacVI-RADS) in which a score of 1 (very high likelihood) to 5 (very low likelihood) is given based on the likelihood of no residual disease after NAC [29]. According to nacVI-RADS, the same negative MRI is given a score of 1 (*very high* likelihood of no residual disease) if there was no perivesical invasion on the pretreatment baseline MRI; however, if there was perivesical invasion present at baseline, then it is given a score of 2 (*high* likelihood of no residual disease). We were unable to confirm this as many of the patients in our study did not have a baseline pretreatment MRI.

As it has been well documented through prior trials, pathological complete response (i.e., no residual disease on cystectomy specimens after NAC) was also associated with better long-term outcomes in patients with MIBC who have received NAC (HR = 4.99, 95% CI 1.50–16.60; p = 0.004). Nevertheless, the main limitation of pathological assessment of post-NAC cystectomy specimens (at least in the point of view of considering the possibility of bladder-sparing approaches) is that this information can only be obtained after performing the completion cystectomy [7]. We believe that the results of our study provide rationale for MRI being part of post-NAC assessment in patients with MIBC. Nevertheless, we do not believe that MRI should be used standalone as the diagnostic performance of MRI was not perfect for predicting absence of residual disease (AUC = 0.76). It may be necessary to combine MRI with other conventional and novel modalities such as cystoscopy, urine cytology, liquid biopsy, and molecular markers [30, 31]. Such a combination of multiple complementary modalities would enable the dream of personalized therapy – providing the option of bladder-sparing approaches to patients who have a good response after NAC (e.g., complete response) and is predicted not to recur or metastasize – become a reality.

Our study had a few limitations. First, the retrospective nature and small number of patients introduces an inherent selection bias. This is partly due to the fact that MRI for response assessment (or as a matter of fact for assessment in any clinical setting) in bladder cancers has not yet been widely adopted compared with other pelvic tumors such as prostate and cervical cancers. However, with the increasing interest in using bladder MRI for assessment of muscle invasion and treatment response, it is anticipated that we will gain more experience in the near future. Additionally, our study included substantially greater number of patients (N = 61) compared with most of the earlier preliminary studies investigating MRI in a similar manner (range, N = 10–48) [5, 19, 20, 29, 32, 33]. Therefore, future studies with larger number of patients conducted in a prospective manner may be able to confirm whether BP-MRI is in fact superior to MP-MRI for evaluating residual tumor on MRI after neoadjuvant chemotherapy in patients with MIBC. Second, patients received various types of systemic treatments, including different chemotherapeutic regimen and different number of cycles. However, this represents the real-world evidence as it is often that patients may not be able to tolerate full regimens of chemotherapy and reflecting the increasing greater number of novel therapeutic agents being developed. Moreover, the timing of post-NAC MRI was not uniform (as it was not performed as part of a clinical trial) and was relatively short (median of 14 days; IQR 7–23), which may have been a potentially source for decreased sensitivity and specificity of MRI for determining residual cancer. Therefore, in order to better understand the diagnostic performance and prognostic role of MRI in the setting of NAC in patients with MIBC, and how information from such can be used to approach bladder-sparing approaches, we will need to incorporate MRI as part of standard assessment modality in clinical trials. Third, inter-reader agreement was not assessed as part of this study. However, it has been shown that in general there is good agreement between radiologist for interpreting MRI for bladder cancer both before and after treatment [29, 34]. Fourth, there is a possibility that interpretation of DCE MRI was influenced by the other sequences (T2WI and DWI). To capture the true extent of which DCE contributes to the MRI interpretation of bladder tumors after NAC, further studies may attempt comparison between BP- and MP-MRI in a randomized fashion.

## Conclusion

BP-MRI was more accurate than MP-MRI, showing a moderately high specificity for determining the presence of residual disease at cystectomy in patients with MIBC that underwent NAC. Furthermore, a negative MRI was associated with better outcomes after cystectomy, providing useful information in addition to pathological assessment of cystectomy specimens. If our findings are further validated, MRI may be used as an additional tool for assessing treatment response and prognostication in MIBC and potential help identify potential candidates for bladder-sparing approaches.

### Electronic Supplementary Material

Below is the link to the electronic supplementary material.

**Supplementary Material 1**: Figure 1. Receiver operating characteristic (ROC) analysis comparing diagnostic performance of biparametric and multiparametric MRI for detecting residual muscle-invasive bladder cancer (MIBC) on cystectomy specimens after neoadjuvant chemotherapy. No statistically significant difference was seen between biparametric and multiparametric MRI with corresponding areas under the ROC of of 0.79 (95% confidence interval [CI], 0.66–0.88) and 0.71 (95% CI, 0.59–0.82), respectively. Using MRI scores of 4–5 as positive, sensitivity and specificity were 82.1% (95% CI, 63.1–93.9) and 72.7% (95% CI, 54.5–86.7) for biparametric MRI and 75.0% (95% CI, 55.1–89.3) and 45.5% (95% CI, 28.1–63.7) for multiparametric MRI.

**Supplementary Material 2**: Figure 2. Kaplan-Meier survival curves for disease-free survival (DFS) in patients who received neoadjuvant chemotherapy followed by cystectomy stratified to (A) muscle-invasive bladder cancer (MIBC) vs non-MIBC on cystectomy specimen and (B) according to ypT stage (no residual tumor, non-MIBC, and MIBC).(A) DFS estimates were significantly worse in patients with MIBC after NAC (hazard ratio [HR] = 5.77, 95% confidence interval [CI] 2.43–13.68, p < 0.001). (B) Overall, there were significant differences in DFS stratified to ypT stage (p < 0.001). While patients with MIBC had significantly worse DFS than those without residual tumor (HR = 7.86, 95% CI 2.32–26.68, p < 0.001) and compared with those with non-MIBC (HR = 4.23, 95% CI 1.45–12.36, p = 0.004), patients with non-MIBC did not show significantly worse DFS compared with those with no residual tumor (HR = 1.86, 95% CI, 0.41–8.35, p = 0.277).

## Data Availability

The datasets during and/or analyzed during the current study available from the corresponding author on reasonable request.

## Abbreviations

AUC: area under the curve
BP: biparametric
CT: computed tomography
DCE: dynamic contrast-enhanced
DFS: disease-free survival
DWI: diffusion-weighted imaging
EMR: electronic medical records
HR: hazard ratio
IV: intravenous
MIBC: muscle-invasive bladder cancer
MP: multiparametric
MRI: magnetic resonance imaging
NAC: neoadjuvant chemotherapy
nacVI-RADS: neoadjuvant chemotherapy Vesical Imaging and Reporting Data System
ROC: receiver operating curve
TUR: transurethral resection
T1WI: T1-weighted imaging
T2WI: T2-weighted imaging
VI-RADS: Vesical Imaging and Reporting Data System

## References

[1] Patel VG, Oh WK, Galsky MD (2020). Treatment of muscle-invasive and advanced Bladder cancer in 2020. CA Cancer J Clin.

[2] Advanced Bladder Cancer Overview Collaboration (2005). Neoadjuvant chemotherapy for invasive Bladder cancer. Cochrane Database Syst Rev.

[3] Grossman HB, Natale RB, Tangen CM, Speights VO, Vogelzang NJ, Trump DL (2003). Neoadjuvant chemotherapy plus cystectomy compared with cystectomy alone for locally advanced Bladder cancer. N Engl J Med.

[4] Finnbladder, International Collaboration of Trialists, Medical Research Council Advanced Bladder Cancer Working Party (now the National Cancer Research Institute Bladder Cancer Clinical Studies Group), European Organisation for Research and Treatment of Cancer Genito-Urinary Tract Cancer Group, Australian Bladder Cancer Study Group, National Cancer Institute of Canada Clinical Trials Group (2011). International phase III trial assessing neoadjuvant cisplatin, methotrexate, and vinblastine chemotherapy for muscle-invasive Bladder cancer: long-term results of the BA06 30894 trial. J Clin Oncol off J Am Soc Clin Oncol.

[5] Choueiri TK, Jacobus S, Bellmunt J, Qu A, Appleman LJ, Tretter C (2014). Neoadjuvant dose-dense methotrexate, vinblastine, doxorubicin, and cisplatin with pegfilgrastim support in muscle-invasive urothelial cancer: pathologic, radiologic, and biomarker correlates. J Clin Oncol off J Am Soc Clin Oncol.

[6] Koga F, Yoshida S, Kawakami S, Kageyama Y, Yokoyama M, Saito K (2008). Low-dose chemoradiotherapy followed by partial or radical cystectomy against muscle-invasive Bladder cancer: an intent-to-treat survival analysis. Urology.

[7] Petrelli F, Coinu A, Cabiddu M, Ghilardi M, Vavassori I, Barni S (2014). Correlation of pathologic complete response with survival after neoadjuvant chemotherapy in Bladder cancer treated with cystectomy: a meta-analysis. Eur Urol.

[8] Kundra V, Silverman PM (2003). Imaging in oncology from the University of Texas M. D. Anderson Cancer Center. Imaging in the diagnosis, staging, and follow-up of cancer of the urinary bladder. AJR Am J Roentgenol.

[9] Choi SJ, Park KJ, Lee G, Kim MH, Kim JK (2020). Urothelial phase CT for assessment of pathologic complete response after neoadjuvant chemotherapy in muscle-invasive Bladder cancer. Eur J Radiol.

[10] Woo S, Panebianco V, Narumi Y, Del Giudice F, Muglia VF, Takeuchi M (2020). Diagnostic performance of Vesical Imaging Reporting and Data System for the prediction of muscle-invasive Bladder Cancer: a systematic review and Meta-analysis. Eur Urol Oncol.

[11] Del Giudice F, Flammia RS, Pecoraro M, Moschini M, D’Andrea D, Messina E (2022). The accuracy of Vesical Imaging-Reporting and Data System (VI-RADS): an updated comprehensive multi-institutional, multi-readers systematic review and meta-analysis from diagnostic evidence into future clinical recommendations. World J Urol.

[12] Woo S, Suh CH, Kim SY, Cho JY, Kim SH, Moon MH (2018). Head-to-head comparison between Biparametric and multiparametric MRI for the diagnosis of Prostate Cancer: a systematic review and Meta-analysis. AJR Am J Roentgenol.

[13] Bricio TGM, Gouvea GL, Barros RV, Chahud F, Elias J, Reis RB (2022). What is the impact of dynamic contrast-enhancement sequence in the Vesical Imaging, Reporting and Data System (VI-RADS)? A subgroup analysis. Cancer Imaging off Publ Int Cancer Imaging Soc.

[14] Manganaro L, Anastasi E, Porpora MG, Vinci V, Saldari M, Bernardo S (2015). Biparametric Magnetic Resonance Imaging as an Adjunct to CA125 and HE4 to improve characterization of large ovarian masses. Anticancer Res.

[15] Woo S, Ghafoor S, Das JP, Gangai N, Goh AC, Vargas HA (2022). Plasmacytoid urothelial carcinoma of the bladder: MRI features and their association with survival. Urol Oncol.

[16] Das JP, Woo S, Ghafoor S, Andrieu PIC, Ulaner GA, Donahue TF (2022). Value of MRI in evaluating urachal carcinoma: a single center retrospective study. Urol Oncol Semin Orig Investig.

[17] Necchi A, Bandini M, Calareso G, Raggi D, Pederzoli F, Farè E (2020). Multiparametric Magnetic Resonance Imaging as a Noninvasive Assessment of Tumor Response to Neoadjuvant Pembrolizumab in muscle-invasive Bladder Cancer: preliminary findings from the PURE-01 study. Eur Urol.

[18] Wang Hjun, Pui MH, Guo Y, Yang D, Pan B, tao (2014). Zhou X Hui. Diffusion-weighted MRI in bladder carcinoma: the differentiation between Tumor recurrence and benign changes after resection. Abdom Imaging.

[19] Yoshida S, Koga F, Kawakami S, Ishii C, Tanaka H, Numao N (2010). Initial experience of diffusion-weighted magnetic resonance imaging to assess therapeutic response to induction chemoradiotherapy against muscle-invasive Bladder cancer. Urology.

[20] Donaldson SB, Bonington SC, Kershaw LE, Cowan R, Lyons J, Elliott T (2013). Dynamic contrast-enhanced MRI in patients with muscle-invasive transitional cell carcinoma of the bladder can distinguish between residual tumour and post-chemotherapy effect. Eur J Radiol.

[21] Wibmer AG, Nikolovski I, Chaim J, Lakhman Y, Lefkowitz RA, Sala E (2022). Local extent of Prostate Cancer at MRI versus Prostatectomy Histopathology: associations with Long-term oncologic outcomes. Radiology.

[22] Iyer G, Balar AV, Milowsky MI, Bochner BH, Dalbagni G, Donat SM (2018). Multicenter prospective phase II trial of Neoadjuvant dose-dense Gemcitabine Plus Cisplatin in patients with muscle-invasive Bladder Cancer. J Clin Oncol off J Am Soc Clin Oncol.

[23] Woo S, Kim SY, Cho JY, Kim SH (2016). Preoperative evaluation of Prostate Cancer aggressiveness: using ADC and ADC ratio in determining Gleason Score. AJR Am J Roentgenol.

[24] Woo S, Suh CH, Kim SY, Cho JY, Kim SH (2017). Diagnostic performance of DWI for differentiating high- from low-Grade Clear Cell Renal Cell Carcinoma: a systematic review and Meta-analysis. AJR Am J Roentgenol.

[25] Ahmed SA, Taher MGA, Ali WA, Ebrahem MAES (2021). Diagnostic performance of contrast-enhanced dynamic and diffusion-weighted MR imaging in the assessment of Tumor response to neoadjuvant therapy in muscle-invasive Bladder cancer. Abdom Radiol N Y.

[26] Bandini M, Calareso G, Raggi D, Marandino L, Colecchia M, Gallina A (2021). The Value of Multiparametric Magnetic Resonance Imaging Sequences to assist in the decision making of muscle-invasive Bladder Cancer. Eur Urol Oncol.

[27] Aslan S, Cakir IM, Oguz U, Bekci T, Demirelli E (2022). Comparison of the diagnostic accuracy and validity of biparametric MRI and multiparametric MRI-based VI-RADS scoring in Bladder cancer; is contrast material really necessary in detecting muscle invasion?. Abdom Radiol N Y.

[28] Thompson HM, Bates DDB, Pernicka JG, Park SJ, Nourbakhsh M, Fuqua JL et al. MRI Assessment of Extramural Venous Invasion before and after total neoadjuvant therapy for locally advanced rectal Cancer and its Association with Disease-Free and overall survival. Ann Surg Oncol. 2023. 10.1245/s10434-023-13225-9 PMC10394736 36964328

[29] Pecoraro M, Del Giudice F, Magliocca F, Simone G, Flammia S, Leonardo C (2022). Vesical Imaging-Reporting and Data System (VI-RADS) for assessment of response to systemic therapy for Bladder cancer: preliminary report. Abdom Radiol N Y.

[30] Wu J, Xie RY, Cao CZ, Shang BQ, Shi HZ, Shou JZ. Disease Management of Clinical Complete Responders to Neoadjuvant Chemotherapy of Muscle-Invasive Bladder Cancer: A Review of Literature. Front Oncol [Internet]. 2022 [cited 2023 May 4];12. Available from: https://www.ncbi.nlm.nih.gov/pmc/articles/PMC9043546/. 10.3389/fonc.2022.816444 PMC9043546 35494010

[31] Iyer G, Ballman KV, Atherton PJ, Murray K, Kwok Y, Steen PD (2022). A phase II study of gemcitabine plus cisplatin chemotherapy in patients with muscle-invasive Bladder cancer with bladder preservation for those patients whose tumors harbor deleterious DNA damage response (DDR) gene alterations (Alliance A031701). J Clin Oncol.

[32] Hafeez S, Koh M, Jones K, Ghzal AE, D’Arcy J, Kumar P (2022). Diffusion-weighted MRI to determine response and long-term clinical outcomes in muscle-invasive Bladder cancer following neoadjuvant chemotherapy. Front Oncol.

[33] Chakiba C, Cornelis F, Descat E, Gross-Goupil M, Sargos P, Roubaud G (2015). Dynamic contrast enhanced MRI-derived parameters are potential biomarkers of therapeutic response in bladder carcinoma. Eur J Radiol.

[34] Del Giudice F, Pecoraro M, Vargas HA, Cipollari S, De Berardinis E, Bicchetti M (2020). Systematic review and Meta-analysis of Vesical Imaging-Reporting and Data System (VI-RADS) Inter-observer Reliability: an added value for muscle invasive Bladder Cancer detection. Cancers.

