# Supplementary material for: Evaluating residual tumor after neoadjuvant chemotherapy for muscle-invasive urothelial bladder cancer: diagnostic performance and outcomes using biparametric vs. multiparametric MRI
Source: Cancer Imaging. 2023 Nov 14;23:110. doi: 10.1186/s40644-023-00632-0 (PMC10644594; doi:10.1186/s40644-023-00632-0)

**Supplementary Figure 1.** Receiver operating characteristic (ROC) analysis comparing diagnostic performance of biparametric and multiparametric MRI for detecting residual muscle-invasive bladder cancer (MIBC) on cystectomy specimens after neoadjuvant chemotherapy. No statistically significant difference was seen between biparametric and multiparametric MRI with corresponding areas under the ROC of of 0.79 (95% confidence interval [CI], 0.66–0.88) and 0.71 (95% CI, 0.59–0.82), respectively. Using MRI scores of 4–5 as positive, sensitivity and specificity were 82.1% (95% CI, 63.1–93.9) and 72.7% (95% CI, 54.5–86.7) for biparametric MRI and 75.0% (95% CI, 55.1–89.3) and 45.5% (95% CI, 28.1–63.7) for multiparametric MRI.


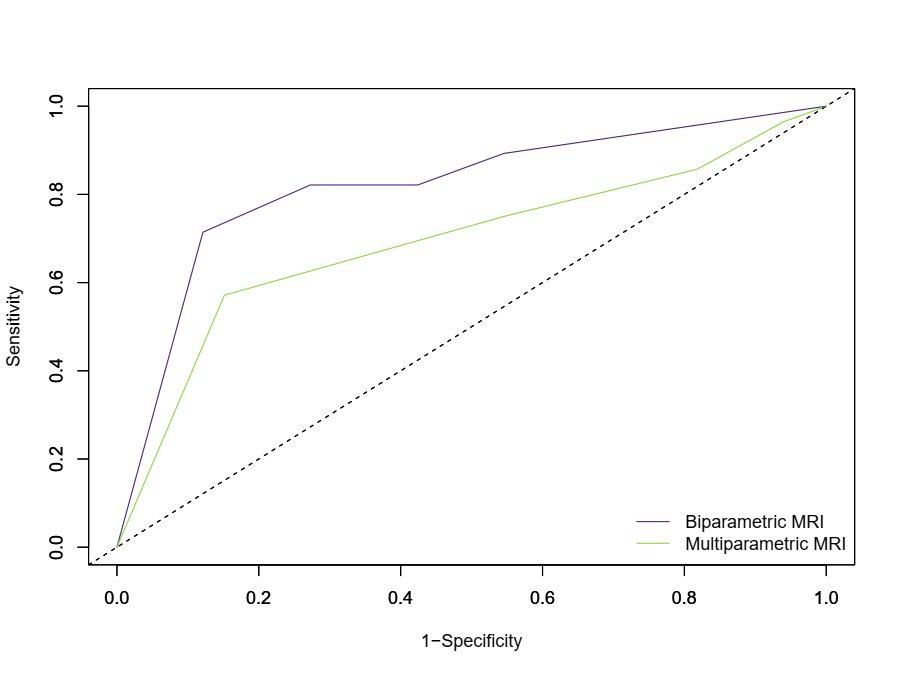

Supplement: Supplementary file 1 — Supplementary Material 1: Figure 1. Receiver operating characteristic (ROC) analysis comparing diagnostic performance of biparametric and multiparametric MRI for detecting residual muscle-invasive bladder cancer (MIBC) on cystectomy specimens after neoadjuvant chemotherapy. No statistically significant difference was seen between biparametric and multiparametric MRI with corresponding areas under the ROC of of 0.79 (95% confidence interval [CI], 0.66–0.88) and 0.71 (95% CI, 0.59–0.82), respectively. Using MRI scores of 4–5 as positive, sensitivity and specificity were 82.1% (95% CI, 63.1–93.9) and 72.7% (95% CI, 54.5–86.7) for biparametric MRI and 75.0% (95% CI, 55.1–89.3) and 45.5% (95% CI, 28.1–63.7) for multiparametric MRI. [file 40644_2023_632_MOESM1_ESM.docx]
