# Supplementary material for: Evaluating residual tumor after neoadjuvant chemotherapy for muscle-invasive urothelial bladder cancer: diagnostic performance and outcomes using biparametric vs. multiparametric MRI
Source: Cancer Imaging. 2023 Nov 14;23:110. doi: 10.1186/s40644-023-00632-0 (PMC10644594; doi:10.1186/s40644-023-00632-0)

**Supplementary Figure 2.** Kaplan-Meier survival curves for disease-free survival (DFS) in patients who received neoadjuvant chemotherapy followed by cystectomy stratified to (A) muscle-invasive bladder cancer (MIBC) vs non-MIBC on cystectomy specimen and (B) according to ypT stage (no residual tumor, non-MIBC, and MIBC).(A) DFS estimates were significantly worse in patients with MIBC after NAC (hazard ratio [HR] = 5.77, 95% confidence interval [CI] 2.43–13.68, p <0.001). (B) Overall, there were significant differences in DFS stratified to ypT stage (p <0.001). While patients with MIBC had significantly worse DFS than those without residual tumor (HR = 7.86, 95% CI 2.32–26.68, p <0.001) and compared with those with non-MIBC (HR = 4.23, 95% CI 1.45–12.36, p = 0.004), patients with non-MIBC did not show significantly worse DFS compared with those with no residual tumor (HR = 1.86, 95% CI, 0.41–8.35, p = 0.277).


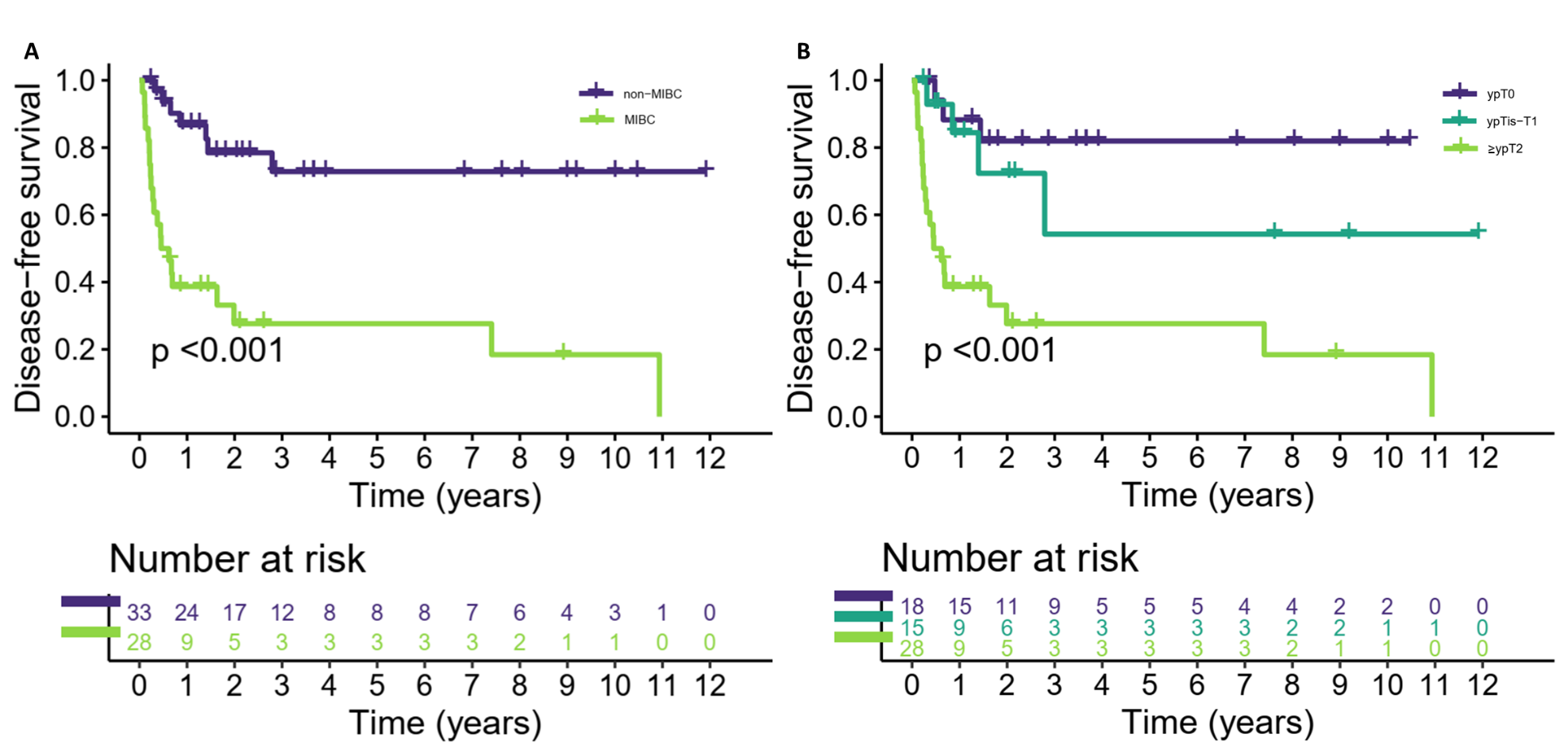

Supplement: Supplementary file 2 — Supplementary Material 2: Figure 2. Kaplan-Meier survival curves for disease-free survival (DFS) in patients who received neoadjuvant chemotherapy followed by cystectomy stratified to (A) muscle-invasive bladder cancer (MIBC) vs non-MIBC on cystectomy specimen and (B) according to ypT stage (no residual tumor, non-MIBC, and MIBC).(A) DFS estimates were significantly worse in patients with MIBC after NAC (hazard ratio [HR] = 5.77, 95% confidence interval [CI] 2.43–13.68, p < 0.001). (B) Overall, there were significant differences in DFS stratified to ypT stage (p < 0.001). While patients with MIBC had significantly worse DFS than those without residual tumor (HR = 7.86, 95% CI 2.32–26.68, p < 0.001) and compared with those with non-MIBC (HR = 4.23, 95% CI 1.45–12.36, p = 0.004), patients with non-MIBC did not show significantly worse DFS compared with those with no residual tumor (HR = 1.86, 95% CI, 0.41–8.35, p = 0.277). [file 40644_2023_632_MOESM2_ESM.docx]
